# Immunoglobulin G4 Cholangiopathy Masquerading as a Klatskin Tumor: An Interesting Case

**DOI:** 10.7759/cureus.17624

**Published:** 2021-08-31

**Authors:** Bianca Varda, Shehroz Aslam, Zaid Ansari, Mital Patel

**Affiliations:** 1 Internal Medicine, Creighton University School of Medicine, St. Joseph's Hospital and Medical Center, Phoenix, USA; 2 Oncology, St. Joseph's Hospital and Medical Center, Phoenix, USA

**Keywords:** cholangiocarcinoma, igg4 disease, klatskin, endoscopy ercp, magnetic resonance cholangiopancreatography (mrcp), bismuth, endoscopic ultrasound (eus), hilar tumour

## Abstract

Immunoglobulin G4 (IgG4) cholangiopathy is used to describe regional biliary involvement in a systemic fibro-inflammatory and infiltrative disease, IgG4-related disease. Occasionally, it tends to present with localized disease leading to an extensive workup to rule out malignancy and infections, especially since IgG4-related disease is an uncommon entity. Herein, we present a case of a 68-year-old male who presented with pruritus and steatorrhea with imaging studies revealing a biliary hilar mass concerning for malignancy. Subsequent extensive testing was inconclusive of malignancy and finally noted to have elevated IgG4 levels as part of a broader workup. The patient was started on prednisone with the resulting improvement in his symptoms and the imaging findings.

## Introduction

Immunoglobulin G4 (IgG4)-related disease is a systemic disease characterized by diffuse lymphoplasmacytic infiltration of IgG4+ plasma cells along with dense storiform fibrosis of the tissues. It generally affects the pancreas with a type 1 autoimmune pancreatitis and the hepatobiliary tree but has the potential to involve any organ in the body. When affecting the biliary system, it tends to be reminiscent of primary sclerosing cholangitis. Rarely, it presents with obstructive jaundice and weight loss as a hilar mass highly suspicious for cholangiocarcinoma or Klatskin tumor. In such scenarios, diagnosis is generally delayed as it requires a high index of suspicion. IgG4 levels are rarely sent as initial tests for evaluation of a hilar mass but ultimately help to confirm the diagnosis in the right clinical setting. Here, we present a case of IgG4 cholangiopathy presenting as a biliary hilar mass concerning for malignancy.

## Case presentation

A 68-year-old male with a history of hepatitis C and remote pancreatitis visualized on prior endoscopic retrograde cholangiopancreatography (ERCP) presumed to be from alcohol use presented with a two-month history of progressive pruritus, steatorrhea, fatigue, and a 20-pound weight loss. Physical examination was notable for scleral icterus, jaundice, and skin abrasions from pruritus. Abdominal examination was benign. Laboratory data revealed obstructive jaundice with a total bilirubin of 8.3 mg/dL, alanine aminotransferase (ALT) of 188 U/L, aspartate aminotransferase (AST) of 78 U/L, and alkaline phosphatase of 596 U/L. Cancer antigen 19‐9 (CA 19-9) was elevated to 53.91 U/ml.

**Figure 1:**
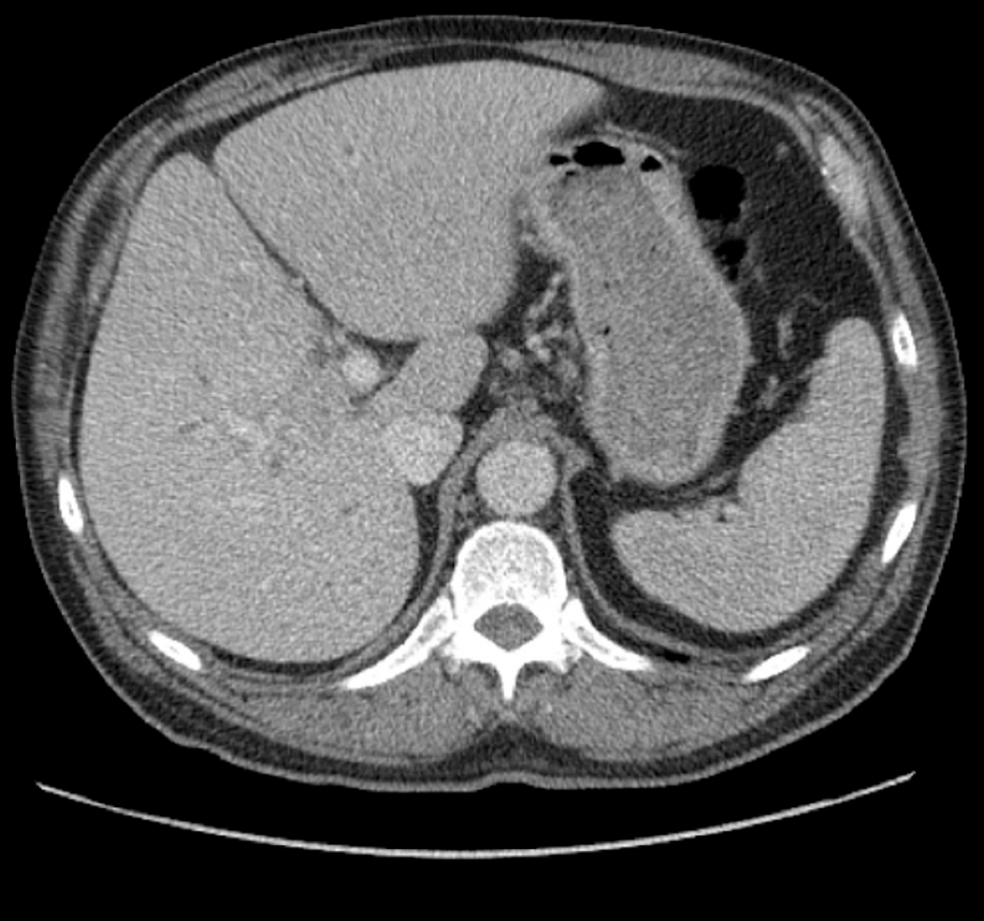
CT showing mild to moderate intrahepatic biliary ductal dilatation with a 4.5 cm x 2.5 cm hilar lesion

**Figure 2:**
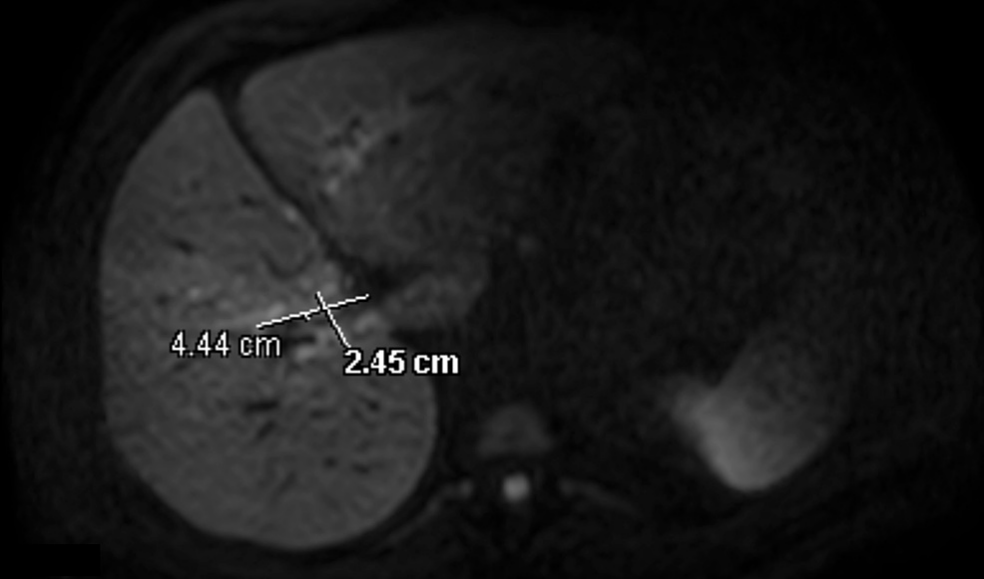
MRCP showing a 4.4 cm x 2.5 cm hypo-enhancing hilar lesion MRCP, Magnetic resonance cholangiopancreatography.

Computed tomography (CT) of the abdomen/pelvis and magnetic resonance cholangiopancreatography (MRCP) showed mild to moderate intrahepatic biliary ductal dilatation with a 4.5 cm x 2.5 cm hilar lesion favoring Klatskin tumor, Bismuth‐Corlette type IV (Figures 1, 2). ERCP showed a common hepatic duct high-grade stricture from a circumferential malignant-appearing mass that was biopsied. Pathology revealed fibrous tissue consistent with mixed inflammation with atypical cell clusters that were suspicious but not diagnostic of malignancy.

He underwent repeat ERCP with endoscopic ultrasound (EUS)-guided biopsy. On EUS, the pancreas was noted to be atrophic and have scattered calcifications, confirming the prior diagnosis of pancreatitis; the pancreatic duct was irregular with mild dilation in the body. Two plastic stents were placed in the right and left intrahepatic ducts during endoscopic retrograde cholangiopancreatography (ERPC). Cholangioscopy showed a malignant-appearing lesion, and repeat biopsies showed atypical appearing cells. Oncology was consulted due to high concerns for a malignancy. Positron emission tomography (PET) scan when obtained showed an avid tumor and lymph nodes in the biliary hilum and diffuse mediastinal and retroperitoneal lymphadenopathy (LAD), which was atypical for hilar cholangiocarcinoma. Hence, differential diagnoses were broadened.

IgG4 level was elevated at 312 mg/dl. In light of unrevealing workup for malignancy, elevated IgG4 levels, cholangitis, and the history of chronic pancreatitis, the patient was started on a trial of steroids for suspected IgG4 cholangiopathy. Prednisone 60 mg daily was given for four weeks for induction followed by an eight-week taper, then stopped. While on prednisone, his symptoms significantly improved confirming the clinical diagnosis. Bilirubin normalized one week after placement of the stents during ERCP. Furthermore, his liver enzymes normalized, and there was a significant downtrend of IgG4 levels (111 mg/dl). The stents were removed after the completion of steroid induction, and liver function test (LFTs) remained normal. Repeat EUS revealed shrinkage of the hilar mass. PET scan also showed decreased lymph node activity, and no vascular involvement was seen. The patient had a recurrence of symptoms when taken off steroids and was later successfully transitioned to azathioprine in the outpatient setting. Currently, two years out, he continues to do well on intermittent prednisone 10 mg with complete resolution of the 4-cm perihilar mass on MR imaging.

## Discussion

IgG4-related disease is a systemic disease characterized by diffuse lymphoplasmacytic infiltration of IgG4+ plasma cells along with dense storiform fibrosis of the tissues. It generally affects the pancreas with a type 1 autoimmune pancreatitis and the hepatobiliary tree but has the potential to involve any organ in the body including salivary glands, lymphadenopathy, and kidneys. IgG4 cholangiopathy, also referred to as IgG4-sclerosing cholangitis (IgG4-SC), is an IgG4-related disease with the involvement of the biliary system and is generally seen with biliary strictures and associated cholangitis. The concomitant presence of autoimmune pancreatitis should raise concerns for IgG4-related disease [1]. While the origin of IgG4 cholangiopathy is unknown, patients often have a history of autoimmune pancreatitis [1]. It is more prevalent in men, with a male to female ratio of 3.3:1, and is most commonly diagnosed at around 48 years of age [1].

Common presenting symptoms are non-specific and include obstructive jaundice, weight loss, or new-onset diabetes [2,3]. The most specific diagnostic indicator of IgG4 cholangiopathy is elevated levels of serum IgG4, which should prompt a close pathologic review [2]. Imaging such as CT and MRI is also used in the diagnostic evaluation of IgG4-SC; however, in rare cases, it was difficult to differentiate between IgG4-related disease and cholangiocarcinoma based on imaging findings alone. One study by Swensson et al. published in 2019 demonstrated that an abrupt cutoff of the common bile duct versus a gradually tapering stricture was found to be a statically significant finding in IgG4-SC not in cholangiocarcinoma [4]. This finding can be used to help differentiate between the two possible diagnoses, but the key diagnostic test is histology. The infiltration of IgG4-positive plasma cells, storiform fibrosis, and obliterative phlebitis is referred to as lymphoplasmacytic sclerosing cholangitis and is diagnostic of IgG4-SC [1]. It is to be noted that infrequently similar histologic findings can be seen in some malignancies and other autoimmune diseases. Hence, IgG4-SC remains a clinical diagnosis supported by histologic, serologic, and imaging features.

The most characteristic feature of IgG4-SC is its dramatic response to steroids and can act as a surrogate confirmatory test [1]. Our patient saw improvement in the treatment with prednisone. Patients often require maintenance doses of steroids. Rituximab is an alternative treatment [2]. Due to the dramatically different treatment options for IgG4 cholangiopathy versus cholangiocarcinoma, it is important to accurately diagnose these conditions and begin the correct treatment promptly. A delay in treatment initiation can in turn lead to fibrosis, organ dysfunction, and even death [5]. Furthermore, differentiating between IgG4-SC and malignancy allows patients to avoid unnecessary treatment and surgeries.

## Conclusions

IgG4 cholangiopathy is the involvement of IgG4-related disease in the biliary system. As the clinical features and diagnostic evaluation between IgG4 cholangiopathy and cholangiocarcinoma overlap, it can be difficult to differentiate between the two, especially when patients present with isolated perihilar masses without any pancreatic involvement. Ultimately, a high index of suspicion prompting evaluation of IgG4 levels supported by critical pathology review is required to make a definitive diagnosis, guide treatment, and reduce the risk of patient morbidity and mortality.
